# Outcome of topiramate in migraine prophylaxis

**DOI:** 10.1097/MS9.0000000000000093

**Published:** 2023-03-24

**Authors:** Etedal Ahmed A. Ibrahim, Wadia Abdalla Balla Elhardallo, Khabab Abbasher Hussien Mohamed Ahmed, Mohammed E. Abdalla Omer, Mohammed Mahmmoud Fadelallah Eljack

**Affiliations:** a Faculty of Medicine, Al-Neelain University; b Faculty of Medicine, University of Khartoum, Khartoum; c Faculty of Medicine and Health Sciences, Gadarif University, Al Qadarif; d Faculty of Medicine and Health Sciences, University of Bakht Alruda, Ad Duwaym, Sudan

**Keywords:** migraine, neurology, prophylaxis, topiramate

## Abstract

**Background:**

Topiramate is an antiepileptic medication originally and one of the first-line drugs for migraine prophylaxis. Herein, we aimed to assess the outcome of topiramate in migraine prophylaxis by evaluating the reduction in frequency and/or severity of attacks and addressing the most common adverse effects associated with it.

**Methods:**

A descriptive, prospective hospital-based study was conducted at Ibrahim Malik Hospital, National Center of Neurological Disease and Sciences from October 2018 to May 2019. A comprehensive, structural, close-ended questionnaire was used to collect data on demographics, clinical, risk factors, treatment, side effects, and outcome.

**Results:**

This study covered 32 study participants; the mean age was 33±10 years, with a female predominance of 27 (84%). Nearly, half of them 15 (47%) migraine triggered by weather changes, and 13 (41%) had menstruation. About 17 (53%) was suffering from headache more than 24 months and most of them 26 (81%) used over-the-counter medications for acute pain headache. The mean frequency of attacks per month was reduced from 6.1 baselines to 3.2, in the severity means was 6.9 turns to 5. Reduction in frequency of attacks there was significant in both number and severity (*P*<0.001) with no significant difference in 50 and 100 mg doses. Concerning adverse effects, 5 (15.6%) did not complain of any, more than a third 12 (38%) experienced weight loss, 7 (22%) both abdominal/gastrointestinal symptoms and dizziness, 5 (16%) mood changes, 4 (13%) both paresthesia and decreased memory, 3 (9%) both anorexia and sleepiness.

**Conclusion:**

Topiramate is effective in reducing headache frequency and reasonably well tolerated in adult Sudanese patients with episodic migraine. This may provide good evidence to support its use in routine clinical management.

HIGHLIGHTS

- Migraine or migraine patients are three times more likely to develop depression than healthy people.
- Topiramate is an antiepileptic medication originally and one of the first-line drugs for migraine prophylaxis.
- We found that’s topiramate effective in migraine prophylaxis with good tolerability and few adverse effects.

## Introduction

Headache is one of the most common diseases of the nervous system. It is estimated that nearly half of the adult population has had at least one headache in the past year. As well as social burdens such as pain, disability, poor quality of life and financial costs. For a few people, a headache itself is a symptom of pain and weakness. It may be a primary disease, namely migraine, tension headache, and cluster headache. Headaches can also be caused by a long list of other diseases or secondary to other diseases, the most common of which is drug abuse headache^1^.

Headache is one of the most common reasons for neurological presentation in sub-Saharan Africa being responsible for 31.9% of visits to Outpatient Neurology Clinics. According to the WHO, 1.7–4% of the adult population of the world has headaches on 15 or more days every month suggesting that up to 170 million adults worldwide have headaches fitting chronic tension-type criteria, according to the International Classification of Headache Disorders. Headache disease brings a heavy burden to patients, and its severity ranges from low quality of life and economic costs to serious comorbidities such as depression. Migraine or migraine patients are three times more likely to develop depression than healthy people^2^.

Globally, the top three causes of consultation for headaches, in both primary and specialist care, are migraine, tension-type headaches, and a combination of these. Tension-type headache is the most prevalent disorder worldwide, but consultation frequencies overall for migraine and tension-type headache only partially reflect this difference. Migraine is associated with a higher probability (per person affected) than the tension-type headache of consultation for headache, and more so in specialists than in primary care. Primary care physicians, nearly universally, are consulted extra frequently for tension-kind headaches, which nearly without a doubt displays its extra prevalence. Specialists on the other hand see more migraine, probably a reflection of their relative severity (greater individual burden)^3^.

In recent years, there have been advances in the treatment of migraine. New therapies for acute and preventive migraine treatment are being prepared. Migraine can have a major impact on the quality of life and daily life. A moderate reduction in the frequency or severity of migraines can bring significant benefits to individuals. Therefore, a need to evaluate the existing treatments thus this study aims to evaluate the outcome of topiramate in migraine prophylaxis in Sudanese patients.

Our aims of this study were to evaluate the outcome of topiramate in migraine prophylaxis among Sudanese patients at Ibrahim Malik Neurological Center–Khartoum State 2018–19, to address factors that trigger a migraine headache, to assess the efficacy of topiramate in migraine prophylaxis and to determine the most common side effects related to topiramate.

## Methods

### Study design

This is a descriptive, prospective hospital-based study conducted at Ibrahim Malik Hospital, National Center of Neurological Disease and Sciences within the period between October 2018 and May 2019. This hospital is located in Khartoum State. It is considered one of the important secondary referral hospitals in Sudan where education opportunities are provided for medical students, house officers, and registrars. This hospital provides medical care for neurological disease patients referred from all over the surrounding residential and rural areas.

This study was conducted in a line with STROCCS criteria 2021^4^.

### Study population

Patients diagnosed with migraine either male or female over 18 years of age, attending the National Center for Neurological Science (NCNS) outpatient clinics, and receiving topiramate for migraine prophylaxis and who fulfill the inclusion criteria. Those who refused to participate and those who did not complete 3 months of topiramate by the end of the study duration were excluded from the study.

### Sample size

The total coverage method was applied as the study participants’ recruitment technique, about 32 participants were the number of patients who met the criteria and agreed to be involved in the study.

### Data collection

Participants were interviewed before and after using topiramate. Data was collected using a comprehensive, structural, close-ended data collection form composed of the following variables:

1. Sociodemographic data including age, sex, occupation, and marital status.
2. Trigger factors including weather changes, stress, food (cheese, chocolate, and caffeine), menstruation, missing meals, exercise, and computer use.
3. Characteristics of the migraine including duration of attack per year, effect on daily activities such as sleep, relationships, work, eating, and analgesics use.
4. Family history of migraine.
5. Topiramate: dose, duration of use, outcome (assessed through number of attacks+pain severity), side effects.

### Data analysis and interpretation

Data were entered, cleaned, and analyzed using SPSS, version 25.0. Descriptive statistics in terms of frequency tables with percentages and graphs. Means and SDs are presented with relevant graphical representations for quantitative data. A *P*-value of 0.05 or less is considered statistically significant. Data represented after analysis in form of univariable tables, cross-tabulation (bivariable tables), figures, and narrative illustration.

### Ethical consideration

Written ethical clearance and approval for conducting this research were obtained from Sudan Medical Specialization Board Ethical Committee. Written permission was obtained from the administrative authority of the NCNS, Khartoum. Study data/information used for research purposes only. Privacy issues are intentionally considered. Participation is voluntary. Any participants have his/her right to stop at any stage. Written informed consent was obtained from all participants.

## Results

This study covered 32 study participants; three-quarters of them 24 (75%) were below 40 years in age; the mean age was 33±10 years, with female predominance by 27 (84%). Nearly a quarter of the participants were housewives 10 (31%), and 6 (19%) were an employee. While nearly half of them 15 (47%) were married. Concerning the factors that trigger their headache, the study found that nearly half of them 15 (47%) triggered by weather changes, 13 (41%) menstruation, 12 (38%) missing meals, 10 (31%) stress, 6 (19%) exercise, 3 (9%) computer use. While food content factors such as 8 (25%) cheese, 5 (16%) caffeine, and 4 (13%) chocolate.

Regarding the family history of migraine headaches among the participants; only 13 (41%) of them have a positive family history. In more detailed history about the characteristics of headache; our study found more than half of the participants 17 (53%) was suffering from headache more than 24 months, 7 (22%) 12–24 months, 6 (19%) 6–12 months, and the rest 2 (6%) less than 6 months. Most of them 26 (81%) used over-the-counter medications for acute pain headaches. While there are many daily activities inhibited by migraine pain, the most common was affected in almost three-quarters of them 23 (72%) sleep, followed by 17 (53%) relationships, 16 (50%) both work and eating.

Regarding details of topiramate dosage, the study found that 25 (78%) were on 50 mg daily, and 7 (22%) were on 100 mg daily. While their period of topiramate usage was distributed as follows: 10 (31%) 3 months, 9 (28%) 6 months, 7 (22%) 4 months, and 6 (19%) 5 months.

The overall reduction rate in frequency was 48%, while the mean frequency of attacks per month was 6.1 reduced to 3.2, in the severity mean was 6.9 turned to 5. Reduction in attacks was as follows a reduction of at least 50% in 17 (53%), frequency reduction of 25–49% in 13 (41%), and only in 2 (6%) the reduction was less than 25%. Paired samples test used for the difference in frequency of attacks was *P*-value less than 0.000 and for severity before and after topiramate also (*P*<0.000) indicating that topiramate is effective in migraine prophylaxis.

In this study, cross-tabulation was done to assess the possible association between the reduction in the frequency of migraine attacks and both topiramate dose and duration of use and the (*P*>0.05).

Concerning adverse effects by study participants due to topiramate; more than a third, 12 (38%) experienced weight loss, 7 (22%) both abdominal/gastrointestinal symptoms and dizziness, 5 (16%) mood changes, 4 (13%) both paresthesia and decreased memory, 3 (9%) both anorexia and sleepiness and only 1 (3%) taste changes. In adverse effect frequency, our study found that 5 (15.6%) did not complain of any adverse effect; while the biggest share 14 (43.8%) have only one adverse effect. Cross-tabulation was done to assess the possible association between the adverse effects of frequency and topiramate dose and duration of use and the (*P*>0.05), detailed in Tables 1 and 2.

**Table 1:** Relation between adverse effects and dose of topiramate (*n*=32)

	Dose of topiramate		
	50 mg	100 mg	Total
Adverse effect			
No adverse effect	5	0	5
One adverse effect	12	2	14
Two adverse effects	4	4	8
Three adverse effects	3	1	4
Four adverse effects	1	0	1
Total	25	7	32

A symptomatic significance (two-sided) *P*=208 (χ^2^ tests).

*P*-value is corresponding to χ^2^ tests – statistical test for the difference between categorical data. *P*<0.05 is significant.

**Table 2:** Relation between adverse effects and duration of using topiramate (*n*=32)

	Duration of the topiramate				
	3 months	4 months	5 months	6 months	Total
Adverse effects					
No adverse effect	2	1	1	1	5
One adverse effect	6	3	3	2	14
Two adverse effects	1	2	1	4	8
Three adverse effects	1	1	1	1	4
Four adverse effects	0	0	0	1	1
Total	10	7	6	9	32

A symptomatic significance (two-sided) *P*=0.854 (χ^2^ tests).

*P*-value is corresponding to χ^2^ tests – statistical test for the difference between categorical data. *P*<0.05 is significant.

## Discussion

This study aimed to assess the outcome of topiramate in migraine prophylaxis in adult Sudanese and covered 32 study participants; three-quarters of them 24 (75%) were below 40 years of age and none above 55, with female predominance by 27 (84%). Similarly, previously described by Vetvik and Macgregor that migraine prevalence initially increases with age, with peak prevalence between 30 and 39 years, followed by a gradual decline over subsequent years in both sexes. After puberty, the sex ratio increases, and women are two to three times more likely to have migraines than men^5^. And by also similar to Martins *et al.* 6 migraineurs were mainly women (81.6%). Just 18.4% were men. Nearly a third of the participants were housewives 10 (31%) and 6 (19%) were an employee. And almost half of them 15 (47%) were married. Corresponding to Ali’s^7^ study finding in a total of 40 Sudanese patients with migraine, 75% as females and 25% as males, the majority of them 18 (45%) were found in the age group 30–39 years and (62%) were married.

Concerning the factors that trigger their headache, the study found that nearly half of them 15 (47%) triggered by weather changes, 13 (41%) menstruation, 12 (38%) missing meals, 10 (31%) stress, 6 (19%) exercise, 3 (9%) computer use. While food content factors such as 8 (25%) cheese, 5 (16%) caffeine, and 4 (13%) chocolate. Similar results were seen in the study by Holzhammer *et al.* 8: The most common trigger factors experienced by the patients were weather (82.5%), stress (66.7%), menstruation (51.4%), and relaxation after stress (50%) As was mentioned by Fukui *et al.* the most common triggers for patients are stress/tension, premature eating, fatigue and lack of sleep in addition to weather, smell, smoke, and light^9^. And while variable results were in a study of triggers factors of migraine which list: at least one dietary trigger, fasting was the most frequent one, followed by alcohol and chocolate. Hormonal factors accounted for 53%, of which the premenstrual period was the most common trigger, 13% of physical activity caused migraine, 2.5% of sexual activity, and 64% of emotional stress as triggers. Overall, 81% cited some sleep problems as triggers. Regarding environmental factors, smells were reported by 36.5%^10^. Regarding the family history of migraine headache among the participants; 13 (41%) of them has a positive family history. The role of heredity in migraine has been a major focus of clinical and research interest for years. Because minimal data are available and contradictory findings as well as methodological inadequacies characterize most of the genetic studies, the genetic assumption is tentative at best^11^.

Migraine without aura seems to be caused by a combination of genetic and environmental factors, while migraine without aura may be primarily or entirely genetic^12^. In more detailed history about the characteristics of headache; our study found more than half of the participants 17 (53%) was suffer from headache more than 24 months, 7 (22%) 12–24 months, 6 (19%) 6–12 months, and the rest 2 (6%) less than 6 months. most of them 26 (81%) used over-the-counter medications for acute pain headaches. While there are many daily activities inhibited by migraine pain, the most commonly affected in almost three-quarters of them is sleep 23 (72%), followed by relationships 17 (53%), and both work and eating 16 (50%). Similarly, a survey has shown that among the millions of Canadians suffering from headaches, adverse effects on relationships with family, friends, and colleagues are common. Headaches also result in significant limitations in activity and lead to avoidance behaviors^13^. And worldwide studies have shown that more than 50% of patients report a disability severe enough to lead to decreased productivity in some of the most important aspects of life–work, school, and home^14^.

Regarding details of topiramate dosage, the study found that 25 (78%) were on 50 mg daily, and 7 (22%) were on 100 mg daily. While their period of topiramate usage was distributed as follows: 10 (31%) 3 months, 9 (28%) 6 months, 7 (22%) 4 months, and 6 (19%) 5 months. According to pharmacological management of migraine guidelines issued in February 2018, topiramate (50–100 mg daily) is recommended as a prophylactic treatment for patients with episodic or chronic migraine and should be used for at least 3 months at the maximum tolerated dose before deciding if it is effective or not^15^.

Prophylaxis aims to reduce the frequency, severity, and duration of migraine attacks and to prevent the development of medication overuse^16^. In our study, the overall reduction rate in frequency was (48%), while the mean frequency of attacks was 6.1 at baseline to 3.2 per month, in the severity mean was 6.9 turn to 5. The result is very close to what Capuano *et al.* were given at the dose of 100 mg/d, in the prophylactic treatment of recorded a significant reduction in the frequency of migraine crises (from 5.26 at baseline to 2.60) in 4 weeks duration^17^. Reduction in attacks was as follow the reduction of at least 50% was in 17 (53%), frequency reduction 25–49% 13 (41%), and only in 2 (6%) reduction was less than 25%.

As migraine can have a considerable impact on quality of life and daily function. Modest improvements in the frequency or severity of migraine headaches may provide considerable benefits. Within trials, a reduction in migraine headache severity and/or frequency of 30–50% is regarded as a successful outcome^15^. Paired samples test used for the difference in frequency of attacks was (*P*<0.000) and for severity before and after topiramate was also (*P*<0.000) indicating that topiramate is effective in migraine prophylaxis. similar to Von Seggern *et al.* 18, a study in which the frequency of headaches at the start of topiramate (baseline) and all subsequent visits up to 24 weeks, declined significantly from baseline to end of treatment (10.684–7.477, respectively; *P*=0.0004).

In adverse effect frequency, our study found that 15.6% did not complain of any adverse effect, while the biggest shares (43.8%) have only one adverse effect. While concerning adverse effects by study participants due to topiramate; more than a third (38%) experienced weight loss, 22% both abdominal/gastrointestinal symptoms and dizziness, 16% mood changes, 4 (13%) both paresthesia and decreased memory, 3 (9%) both anorexia and sleepiness and only 1 (3%) taste changes. Linde and colleagues conducted a systematic review in trials of topiramate against a placebo, seven adverse events (AEs) were reported by at least three studies. These were usually mild and of a nonserious nature. Except for dysgeusia and weight loss, there were no significant differences in the total AE or the incidence of seven specific AEs between placebo and topiramate 50 mg. The frequency of AEs in general and all specific AEs except nausea is significantly higher on Topiramate 100^19^. Moreover, another study revealed that topiramate 100 mg daily was associated with a higher rate of AEs than placebo, although these were mild to moderate.

Side effects include nausea, paresthesia, anorexia, and weight loss. Cognitive side effects are common, vary in severity, are usually dose-dependent, and usually determine drug tolerability. Depression is also a common side effect^15^. In a study done by James Adelman *et al.* in which two groups where included (one group was given placebo and the other one was given topiramate the following were detected: Fatigue (5%), nausea (2%), and difficulty concentrating (2%). Compared with the placebo, the average body weight of patients treated with topiramate was significantly lower^20^.

The study had some limitations; the relatively limited number of study participants (32 study participants from one study area only) may affect negatively the probability of finding more significant outcomes among patients receiving topiramate for migraine in other Sudanese hospitals. Another limitation is follow-up. Some outcomes – such as a long-term outcome or the presence of long-term effects and/or adverse effects – may need to be followed over time for a longer period. So, a long-term prospective cohort follow-up design may be useful for a more detailed description of the confirmatory practices.

## Conclusions

Migraine affects more young females. Migraine pain interferes with many daily activities, including sleep, relationships, work, and eating. Topiramate reduces the frequency and severity of migraine attacks by nearly half, indicating that it is effective in migraine prevention in terms of both frequency and severity.

In terms of side effects, the most common were weight loss, followed by weight loss, abdominal/gastrointestinal symptoms, dizziness, and mood changes, with the least percentage reporting none.

## Recommendations

Increase the awareness of the primary care doctors and general practitioners by the International Health Society criteria and the diagnostic criteria of the International Health Society for a migraine headache to avoid misdiagnosis. Physicians and neurologists should take into their account the established efficacy and safety record topiramate has in the prophylaxis of migraine. Further cohort follow-up – research is highly recommended to assess to long-term outcome of topiramate in migraine, and comparing with other prophylactic medications.

## Sources of funding

This research did not receive any specific grant from funding agencies in the public, commercial, or not-for-profit sectors.

## Authors’ contribution

All authors participated in planning the study, data collection, results, and discussion sections.

## Conflicts of interest disclosure

The authors declare that they have no financial conflict of interest with regard to the content of this report.

## Research registration unique identifying number (UIN)

Not applicable.

## Guarantor

Mohammed Mahmmoud Fadelallah Eljack.

## Provenance and peer review

Not commissioned, externally peer-reviewed.
